# *GRIA* Gene Expression in Schizophrenia: A Participant-Level Meta-Analysis

**DOI:** 10.31083/AP46200

**Published:** 2026-05-21

**Authors:** Adan Baumel, Assif Yitzhaky, Libi Hertzberg

**Affiliations:** ^1^School of Medicine, Faculty of Medical and Health Sciences, Tel Aviv University, 6997801 Tel Aviv, Israel; ^2^Department of Physics of Complex Systems, Weizmann Institute of Science, 7610001 Rehovot, Israel; ^3^Adult Inpatient Ward, Shalvata Mental Health Center, 45100 Hod Hasharon, Israel

**Keywords:** gene expression profiling, organoids, receptors, AMPA, glutamate, schizophrenia

## Abstract

**Background:**

Schizophrenia is a complex mental disorder with an estimated heritability of 80%, yet its underlying pathophysiology remains poorly understood. Emerging evidence implicates the α-amino-3-hydroxy-5-methyl-4-isoxazole propionic acid (AMPA) glutamate receptor—a key player in fast excitatory synaptic transmission, encoded by Glutamate Ionotropic Receptor AMPA Type Subunits 1-4 (*GRIA1-4*)—in the disorder's pathophysiology. However, findings from postmortem brain samples regarding *GRIA1-4* expression have been inconsistent. This study aimed to systematically evaluate the differential expression of *GRIA1-4* genes in schizophrenia by integrating transcriptomic data from postmortem brain tissue and patient-derived cerebral organoids.

**Methods:**

We conducted a participant-level meta-analysis of seven postmortem brain sample datasets (n = 295; 151 schizophrenia, 144 controls) and analyzed an independent organoid dataset (n = 16). Expression differences between schizophrenia and control samples were quantified using Hedges' g under a random-effects model, with heterogeneity assessed using the I^2^ statistic.

**Results:**

Our analysis revealed significant downregulation of all four *GRIA* genes (*GRIA1-4*) in postmortem brain tissue from individuals with schizophrenia, with the effect concentrated in a subgroup of patients. No substantial heterogeneity was attributable to differences in brain regions or measurement platforms. Consistent downregulation of *GRIA1-3* was observed in patient-derived cerebral organoids, which model early neurodevelopmental stages.

**Conclusions:**

Our findings highlight AMPA receptor dysfunction as a potential contributor to schizophrenia pathophysiology in a subgroup of patients, consistent with the broader role of glutamatergic signaling disruption in this disorder. The convergent evidence from postmortem brain tissue and developmental models underscores the need for further investigation of *GRIA* genes as potential biomarkers for patient stratification and as therapeutic targets. While further study is needed to understand the functional consequences of our findings, such insights may inform the development of personalized treatment strategies targeting glutamatergic dysfunction in schizophrenia.

## Main Points

(1) Glutamate Ionotropic Receptor AMPA Type Subunits 1-4 (*GRIA1-4*) expression is significantly reduced in schizophrenia across seven postmortem brain datasets.

(2) A coordinated downregulation of *GRIA1-4* characterizes a distinct molecular subgroup of individuals with schizophrenia.

(3) Similar downregulation of *GRIA1-3* is present in schizophrenia-derived cerebral organoids, supporting developmental involvement.

(4) These findings highlight α-amino-3-hydroxy-5-methyl-4-isoxazole propionic acid (AMPA) receptor signaling as a potential biomarker and therapeutic target.

## 1. Introduction

Schizophrenia is a chronic psychiatric disorder with a lifetime prevalence of about 0.7% [1], characterized by a complex constellation of symptoms that span three primary domains: positive symptoms, such as hallucinations and delusions; negative symptoms, including social withdrawal and flattened affect; and cognitive impairments, including deficits in working memory and verbal learning. Unlike the transient nature of psychosis, these persistent symptomatic clusters, especially cognitive and negative symptoms, drive significant social and occupational disability. Consequently, the disorder imposes a profound socioeconomic and emotional burden on affected individuals, their families, and global healthcare systems [2, 3, 4].

Schizophrenia has a strong genetic component, with heritability estimated at about 80% [5]. Genome-wide association studies (GWAS) have identified more than 290 risk loci, each contributing only a modest increase in risk [6], and together explaining 25–50% of genetic liability [7, 8]. Many of the associated variants are located in noncoding regions, which are enriched with regulatory sequences that influence gene expression rather than protein structure [9]. Therefore, it is essential to examine gene expression patterns and identify genes with differential expression in schizophrenia to better understand the genetic and molecular basis of the disease.

For decades, the dopamine hypothesis has dominated the neurochemical understanding of schizophrenia, proposing that symptoms result from increased dopaminergic activity in the striatum and decreased activity in prefrontal regions [10, 11, 12, 13]. Another hypothesis that has gained significant support in recent decades is the involvement of glutamatergic signaling in schizophrenia. Glutamatergic dysfunction has been associated with both psychotic symptoms and cognitive deficits [14, 15, 16]. This hypothesis is mainly supported by the psychotomimetic effects of noncompetitive N-methyl-D-aspartate (NMDA) receptor antagonists such as ketamine and phencyclidine [17, 18, 19, 20], suggesting that NMDA receptor hypofunction may play a role in symptom development. However, focusing only on NMDA receptor dysfunction might oversimplify the underlying pathophysiology. Clinical trials targeting glutamatergic transmission have shown limited therapeutic benefit [21], indicating that glutamate-related changes probably go beyond receptor binding and involve more extensive synaptic and circuit-level processes.

Glutamate receptors are divided into two main groups: (1) Ionotropic receptors, which respond to synthetic glutamate derivatives like NMDA, AMPA (α-amino-3-hydroxy-5-methyl-4-isoxazole propionic acid), and kainate; and (2) G-protein coupled metabotropic receptors that produce longer-lasting neuromodulatory effects of glutamate [22].

NMDA receptors co-localize with AMPA receptors (AMPARs) at excitatory synapses throughout the brain, where AMPAR activation modulates N-methyl-D-aspartate receptors (NMDARs) function [23, 24]. AMPARs are multimeric structures composed of four subunits, encoded by the Glutamate Ionotropic Receptor AMPA Type Subunits 1-4 (*GRIA1-4*) genes (referred to as *GluR1–4* in earlier literature) [23, 25], which can assemble in multiple homo- or heteromeric combinations, contributing to substantial receptor diversity. AMPARs mediate fast excitatory neurotransmission through Na^+^ influx and subsequent postsynaptic depolarization, which relieves the Mg^2+^ block of the NMDA receptor channel and allows Ca^2+^ entry [23, 26]. NMDARs-dependent long-term potentiation and depression (LTP/LTD), both critical mechanisms underlying learning and memory, rely on the dynamic regulation of AMPAR trafficking [27, 28]. Individuals with schizophrenia experience cognitive impairments, including various learning and memory deficits [29]. It has also been suggested that enhancing AMPA receptor activity could be a practical way to address NMDA receptor hypofunction and the associated cognitive impairments in schizophrenia [26]. Animal studies further highlight the role of AMPARs in behaviors associated with schizophrenia. *GRIA1* knockout mice exhibited behavioral abnormalities linked to schizophrenia, including increased locomotor activity in response to novelty, impaired prepulse inhibition, disorganized social behaviors, and a lack of pleasure responses [30, 31, 32].

At the genetic level, *GRIA* genes are highly conserved among mammals [33]. *GRIA1* was found to be associated with schizophrenia in a GWAS that identified 108 associated loci [34], as well as in a study of a Korean population [35]. Importantly, *GRIA3* was identified as one of 10 genes with ultra-rare variants that were associated with schizophrenia, in a study of the Schizophrenia Exome Sequencing Meta-Analysis (SCHEMA) consortium [36]. Findings regarding the associations of *GRIA2* and *GRIA4* with schizophrenia have been inconsistent across studies [37, 38, 39, 40].

A systematic review of postmortem studies examining AMPA receptor subunits’ expression and binding in patients with schizophrenia reported inconsistent results. This inconsistency was observed across studies investigating AMPA receptor binding, subunit protein expression, and subunit messenger RNA (mRNA) expression [41]. Few studies have observed a reduction in AMPA subunits’ mRNA expression in brain samples from patients with schizophrenia compared to healthy control subjects [42, 43, 44], while others found no significant difference [45, 46], and a few studies reported increased mRNA expression [47, 48]. Few studies have reported a decrease in AMPA expression at the protein level [49, 50], while others did not find any significant difference [51, 52, 53], and some observed increased protein levels [54, 55]. Similarly, findings on AMPA receptor binding were inconsistent. Some studies found reduced receptor binding in brain samples from individuals with schizophrenia [56, 57], whereas others reported no significant differences [44, 58, 59, 60], or increased receptor binding [61, 62].

Preclinical studies demonstrate that positive allosteric modulators, such as AMPAkines, enhance synaptic plasticity and cognitive performance and may potentiate antipsychotic efficacy in the context of glutamatergic dysfunction [63, 64]. Therapeutic modulation of AMPARs has therefore emerged as a promising avenue for treating schizophrenia. However, translation to clinical efficacy has been limited by challenges related to drug potency, subunit selectivity, and tolerability [64, 65].

Brain samples from individuals with schizophrenia are only accessible postmortem, which may not reflect changes that occur during the early stages of the disease. Furthermore, the differential expression observed in postmortem samples might be influenced by confounding factors such as medication and duration of illness. Brain organoid models, created from induced pluripotent stem cells (iPSCs), serve as valuable tools for studying early human brain development in the context of neurodevelopmental disorders. A study using organoids derived from individuals with schizophrenia reported altered expression of genes involved in synaptic and neurodevelopmental processes compared to healthy controls, including downregulation of *GRIA1-3* [65].

Given the important role of glutamate signaling in schizophrenia, the evidence for an association at the genetic level, and the inconsistent findings on *GRIA* gene expression, we systematically compared *GRIA* expression levels in brain samples from patients with schizophrenia and healthy individuals. Following the Preferred Reporting Items for Systematic Reviews and Meta-Analyses (PRISMA) 2020 guidelines [66], we conducted a participant-level meta-analysis of seven publicly available datasets (overall 295 samples). Additionally, we analyzed an organoid dataset, produced from patients with schizophrenia and controls, to explore *GRIA* during early neurodevelopment.

## 2. Methods and Materials

### 2.1 Dataset Identification and Selection

**Fig. 1.**
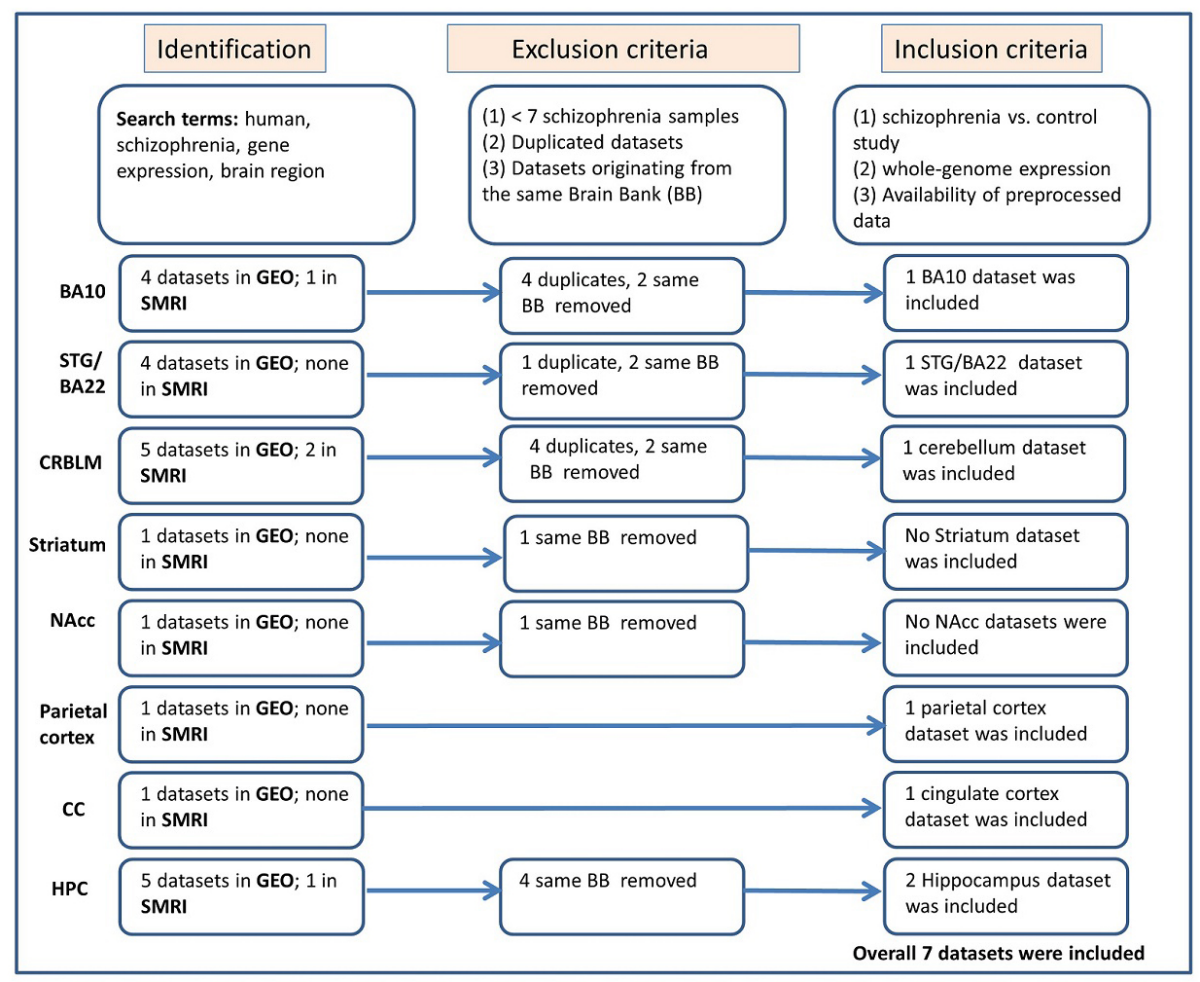
**Diagram illustrating the study identification and selection process conducted in accordance with PRISMA 2020 guidelines**. The flowchart summarizes dataset retrieval, screening stages, and final inclusion criteria. Abbreviations: SMRI, Stanley Medical Research Institute; GEO, Gene Expression Omnibus; CMC, Common Mind Consortium; CC, cingulate cortex; BA, brodmann area; STG, Superior Temporal Gyrus; CRBLM, cerebellum; NAcc, Nucleus Accumbens; HPC, hippocampus; PRISMA, Preferred Reporting Items for Systematic Reviews and Meta-Analyses.

Gene expression datasets were identified through a structured search of the National Center for Biotechnology Information Gene Expression Omnibus (NCBI GEO; https://www.ncbi.nlm.nih.gov/geo/) and the Stanley Medical Research Institute (SMRI) Array Collection (https://www.stanleyresearch.org/brain-research/array-collection/). Searches included the terms schizophrenia, gene expression, human, and brain. Dataset screening and selection procedures followed the PRISMA 2020 guidelines [66], and the full workflow is illustrated in Fig. 1. Studies were eligible if they: (1) included postmortem human brain samples from individuals with schizophrenia and healthy controls, (2) provided normalized expression matrices, and (3) sampled one of the following brain regions: Brodmann area (BA) 10, superior temporal gyrus (BA22), cerebellum, parietal cortex, anterior cingulate cortex, nucleus accumbens, striatum, or hippocampus. Datasets were excluded when they contained overlapping samples obtained from the same brain bank. Study metadata included sample sizes, demographic information, assay platform, postmortem interval (PMI), and tissue pH (Table 1, Ref. [67, 68, 69, 70, 71, 72, 73, 74]). Additional preprocessing steps, normalization, quality control, and outlier removal are reported in the **Supplementary Materials**.

**Table 1.** **Summary of clinical profiles and technical specifications for all datasets used in the meta-analysis**.

Postmortem Brain sample datasets (295 samples)								
Accession	Publication	Brain region; Brain bank	# SZ (151 samples)	# CNT (144 samples)	Platform	Mean Age (standard dev.)	Mean PMI (standard dev.)	Mean pH (standard dev.)
GDS4523	(Maycox *et al*., 2009) [67]	BA10; CCHPC	27	23	HG U133 Plus 2.0	SZ: 73 (15)	SZ: 8.2 (7)	SZ: 6.1 (0.2)
			19M:8F	12M:11F		CNT: 69 (22)	CNT: 10 (4)	CNT: 6.5 (0.3)
						*p* = 0.45	*p* = 0.30	***p* = 8 × 10^-6^**
GSE37981	(Pietersen *et al*., 2014) [68]	STG Pyramid.; HBTRC	9	8	U133 X3P Array	SZ: 67 (20)	SZ: 17 (5)	Not provided
			4M:5F	4M:4F		CNT: 67 (21)	CNT: 18 (3)	
						*p* = 0.99	*p* = 0.71	
GDS1917	(Paz *et al*., 2006) [69]	CRBLM; Maryland	13	14	U133 Plus 2.0 Array	SZ: 46 (12)	SZ: 12.8 (5)	Not provided
			13M:0F	14M:0F		CNT: 43 (10)	CNT: 15.6 (6)	
						*p* = 0.50	*p* = 0.18	
GSE35978	(Chen *et al*., 2013) [70]	Parietal cortex; SMRI	51	45	Gene 1.0 ST Array	SZ: 43 (10);	SZ: 31 (16)	SZ: 6.4 (0.3)
			37M:14F	31M:14F		CNT: 46 (9)	CNT: 27 (12)	CNT: 6.5 (0.3)
						*p* = 0.14	*p* = 0.17	***p* = 0.015**
GSE80655	(Ramaker *et al*., 2017) [71]	ACC; Pritzker	23	24	Illumina HiSeq 2000	SZ: 43 (9);	SZ: 21 (9)	SZ: 6.8 (0.2)
			20M:3F	21M:3F		CNT: 50 (13)	CNT: 22 (7)	CNT: 6.9 (0.1)
						*p* = 0.043	*p* = 0.62	***p* = 0.044**
GSE53987	(Lanz *et al*., 2019) [72]	HPC; Pittsburgh	15	18	HG U133 Plus 2.0	SZ: 46 (9);	SZ: 19 (7)	SZ: 6.4 (0.3)
			9M:6F	9M:9F		CNT: 48 (11)	CNT: 19 (5)	CNT: 6.6 (0.2)
						*p* = 0.49	*p* = 0.99	*p* = 0.055
GSE138082	(Perez *et al*., 2021) [73]	HPC CA3; Dallas	13	12	Illumina HiSeq 2500	SZ: 54 (11);	SZ: 23 (7)	Not provided
			9M:4F	9M:3F		CNT: 56 (9)	CNT: 20 (4)	
						*p* = 0.59	*p* = 0.20	
Organoid samples dataset (16 samples)								
Accession	Publication	Samples type	# SZ (8 samples)	# CNT (8 samples)	Platform			
GSE133534	(Kathuria *et al*., 2020) [74]	iPSC derived cerebral organoids	8 5M:3F	8 6M:2F	Illumina NovaSeq 6000			

#, number of; SZ, schizophrenia; CNT, controls; PMI, postmortem interval; ACC, anterior cingulate cortex; CA3, Cornu Ammonis 3; pyramid., pyramidal; parvalb., parvalbumin; M, males; F, females; Pritzker, Pritzker Neuropsychiatric Disorders Research Consortium; Pittsburgh, Brain Tissue Donation Program at the University of Pittsburgh; Dallas, Dallas Brain Collection; Maryland, Maryland Brain Collection; HBTRC, The Harvard Brain Tissue Resource Center; CCHPC, Charing Cross Hospital Prospective Collection; iPSC, induced pluripotent stem cells; Two-sided *t*-test associated *p*-values are listed for each dataset, for having different (SZ vs. CNT) mean age, PMI, and pH; Statistically significant differences (*p*-value < 0.05) appear in bold.

### 2.2 Postmortem Brain Samples Differential Gene Expression Meta-Analysis

Differential expressions of *GRIA* genes were assessed using an individual participant-level meta-analytic framework. Standardized mean differences between schizophrenia and control groups were quantified using Hedges’ g [75], with positive values representing higher expression among schizophrenia samples and negative values indicating reduced expression. Effect sizes and 95% confidence intervals were estimated using the “metacont” function from the R *meta* package (version 3.6.1, R Foundation for Statistical Computing, Vienna, Austria) [76]. Because the included datasets differed in brain regions, platforms, and preprocessing strategies, pooled effect sizes were calculated using a random-effects model [77], which accounts for both study and between-study variability and improves generalizability [78].

To assess heterogeneity across the datasets included in the meta-analysis, we quantified between-study variability using three complementary measures: Cochran’s Q, I^2^, and τ^2^. Cochran’s Q provides a formal statistical test for heterogeneity by evaluating whether the observed variability in effect sizes exceeds what would be expected by chance alone, based on the weighted sum of squared deviations from the pooled effect size [79]. To quantify the degree of inconsistency that could be attributed to genuine between-study variation rather than chance, the I^2^ (Inconsistency) statistic was calculated, expressing the proportion of total variation due to heterogeneity [80]. Furthermore, the magnitude of the between-study variance was estimated using τ^2^, which provided the essential variance component for the subsequent random-effects model employed in the meta-analysis [81]. Together, these metrics allowed us to evaluate the extent and nature of heterogeneity among the included datasets and to interpret the robustness of the meta-analytic findings.

### 2.3 Analysis of Human iPSCs-Derived Cerebral Organoids of Patients With Schizophrenia

Differential expression of *GRIA* genes was measured in a patient-derived organoid dataset, which was downloaded from the GEO database (GSE133534) and comprised 16 samples (8 from patients with schizophrenia and 8 from controls). Study metadata included sample sizes, sex, and assay platform (Table 1). Additional preprocessing steps, normalization, quality control, and outlier removal are reported in the **Supplementary Materials**. Differential log_2_ expression was calculated using a two-sided *t*-test.

### 2.4 Analysis of Potential Confounding Variables

To assess whether group differences in gene expression could be influenced by clinical or biological covariates, multiple linear regression models were fitted for each dataset using the MATLAB “fitlm” function [82]. Available covariates included age, sex, PMI, tissue pH, and antipsychotic medication history. Schizophrenia diagnosis served as the primary predictor variable. Regression results are reported as *t*-statistics and *p*-values, indicating whether differential expression remained significant after adjustment.

## 3. Results

### 3.1 Meta-Analysis Reveals Reduced GRIA1-4 Expression in Schizophrenia

**Fig. 2.**
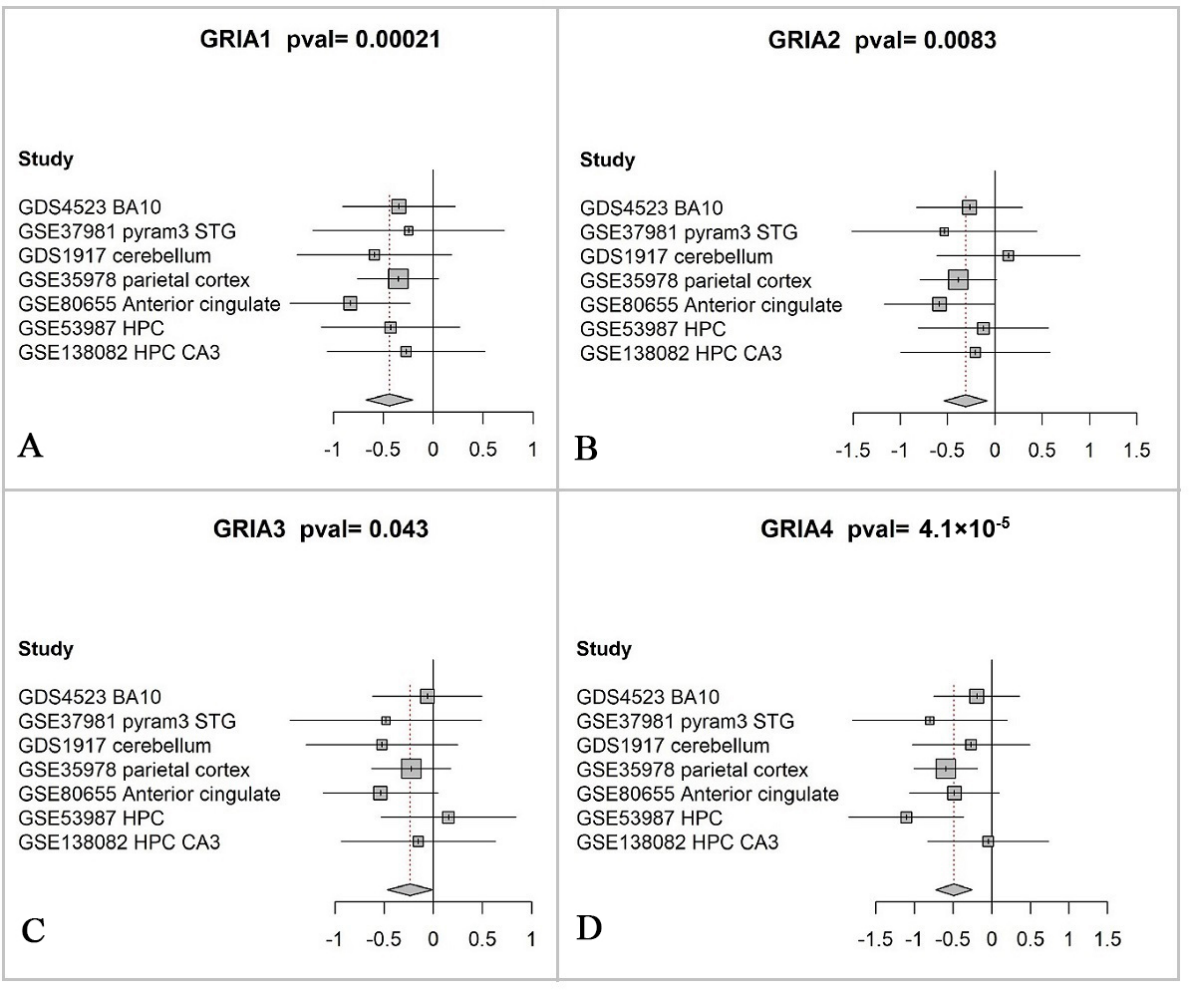
**Reduced expression of *GRIA1-4* in postmortem brain tissue from patients with schizophrenia**. Forest plots show the differences in expression levels of *GRIA1* (A), *GRIA2* (B), *GRIA3* (C), and *GRIA4* (D) between individuals with schizophrenia and healthy controls across all datasets included in the meta-analysis. Forest plots were generated using the meta package in R *meta* package (version 3.6.1) with its built-in plotting function. In each panel, the square symbols represent the standardized mean difference (Hedges’ g) for individual datasets, and their size reflects the relative analytical weight determined largely by sample size. The corresponding 95% confidence intervals are represented by horizontal bars extending from each square. A vertical line indicates the point of zero difference. The standardized mean difference is positive (negative) if the expression is higher (lower) in schizophrenia compared to the control group. The center of the diamond represents the overall difference across all studies, and its width represents the corresponding 95% confidence interval. The associated *p*-values are reported in the figure titles.

A meta-analysis of GRIA1-4 differential expression was conducted, revealing significant downregulation of all four genes in individuals diagnosed with schizophrenia relative to control subjects (Fig. 2; Table 2).

**Table 2.** **Glutamate Ionotropic Receptor AMPA (*GRIA)* genes meta-analysis results**.

	Gene symbol	Random effects Hedges	Lower	Upper	*p*-value	τ^2^	I^2^	Q	Q *p*-value
1	*GRIA1*	–0.44	–0.67	–0.21	0.00021	0	0	2.4	0.88
2	*GRIA2*	–0.31	–0.54	–0.08	0.0083	0	0	2.9	0.82
3	*GRIA3*	–0.24	–0.47	–0.01	0.043	0	0	3.5	0.75
4	*GRIA4*	–0.49	–0.72	–0.26	4.10 × 10^-5^	0	0	5.9	0.43

The standardized mean difference (Hedges’ g, random effects model) is negative (bluish) when expression is lower in schizophrenia compared to controls. The color intensity is proportional to the Hedges’ g value. The lower and upper 95% confidence interval limits are given in the 4th and 5th columns. The associated *p*-values are given in the 6th column. Heterogeneity measures - I^2^, τ^2^, and Cochran’s Q test and its associated *p*-value are given in columns 7th–10th. AMPA, α-amino-3-hydroxy-5-methyl-4-isoxazole propionic acid.

### 3.2 GRIA1-3 are Downregulated in Human iPSCs-Derived Cerebral Organoids of Patients With Schizophrenia

**Fig. 3.**
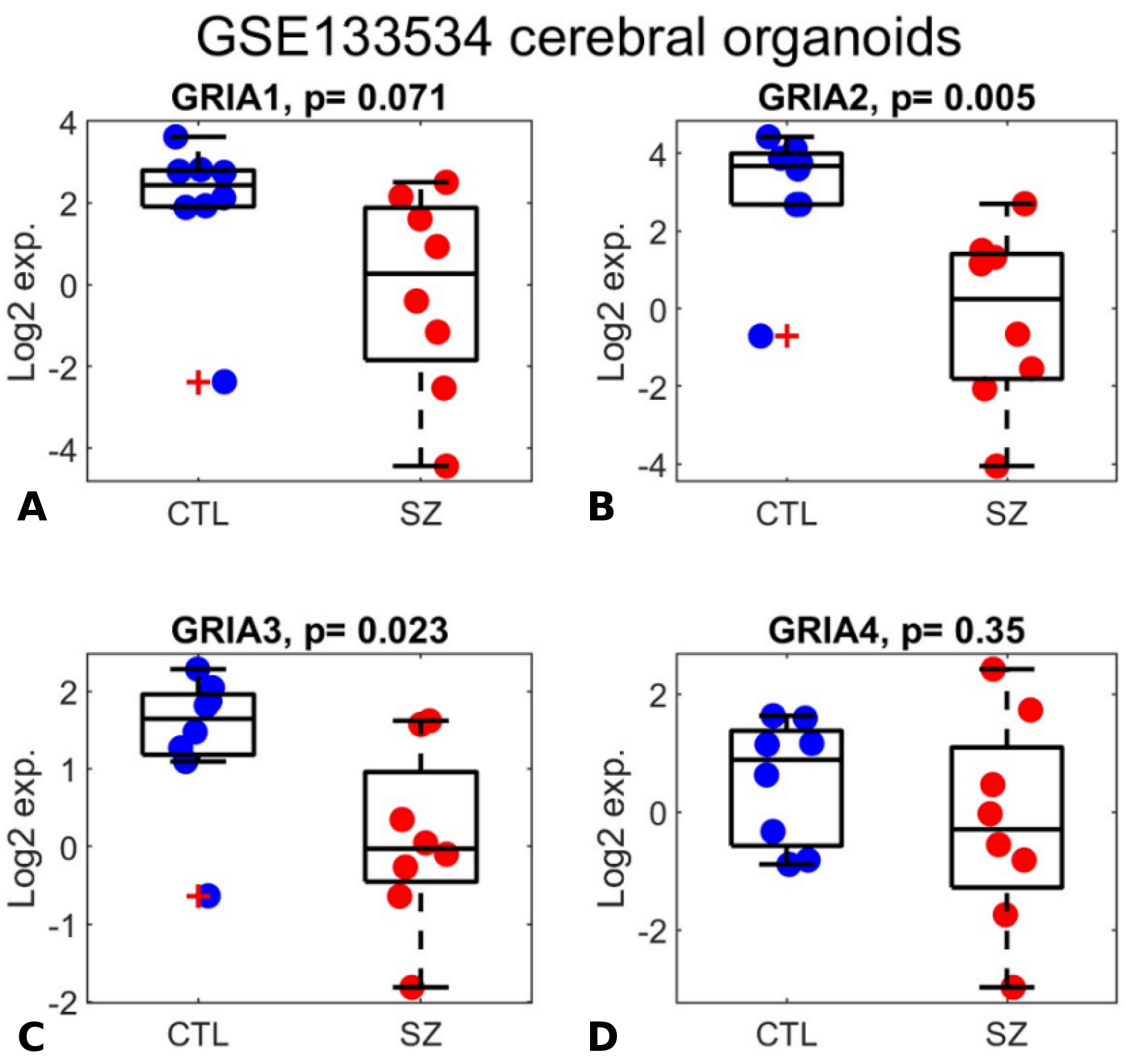
***GRIA1-3* expression is downregulated in human iPSC-derived cerebral organoids from individuals with schizophrenia**. (A–D) Box plots display expression levels for *GRIA1* (A), *GRIA2* (B), *GRIA3* (C), and *GRIA4* (D). The *y*-axis represents log₂-transformed expression values. Each dot corresponds to the log₂ expression level of an individual in the GSE133534 cerebral organoid dataset, including healthy controls (blue dots; labeled “CTL”) and individuals with schizophrenia (red dots; labeled “SZ”). The central mark indicates the median, while the bottom and top edges of each box represent the 25th and 75th percentiles, respectively. Two-sided *t*-test *p*-values are reported in the subplot titles.

**Fig. 4.**
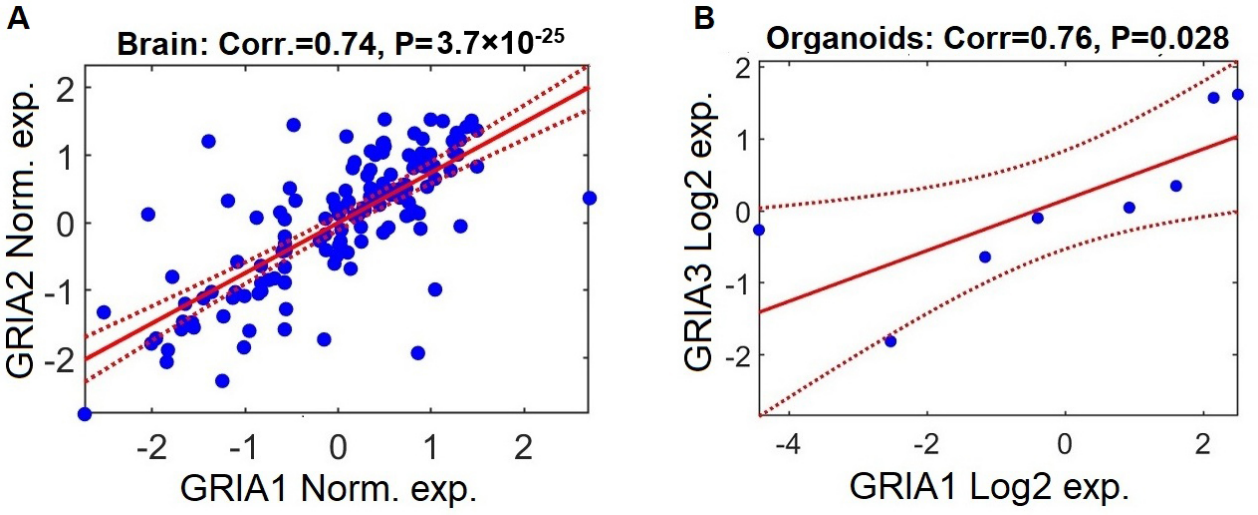
***GRIA* gene expression patterns are positively correlated in brain samples and cerebral organoids from individuals with schizophrenia**. (A) Scatter plot demonstrating the relationship between log_2_-normalized expression levels of *GRIA2* and *GRIA1* in postmortem brain samples from individuals with schizophrenia, based on aggregated data from the seven datasets included in the meta-analysis. Each data point reflects expression values for a single participant. Normalization was performed independently within each dataset (z-scoring: mean = 0, standard deviation = 1). The solid red line shows the fitted linear regression trajectory, and the dashed red curves represent the 95% confidence interval. (B) Parallel analysis depicting the association between log_2_-normalized expression levels of *GRIA3* and *GRIA1* in schizophrenia-derived cerebral organoids.

*GRIA1-3* were previously shown to be significantly downregulated in schizophrenia in a study of organoids derived from patients, modeling early developmental stages. However, the differential expression of *GRIA4* was not referred to [65]. We analyzed the same organoids gene expression dataset, from the GEO database (GSE133534) and comprised of 16 samples (8 schizophrenia and 8 controls). *GRIA1-3* were indeed downregulated in schizophrenia (Fig. 3; *p* = 0.071, 0.005, 0.023, respectively), while *GRIA4* did not show significant differential expression (*p* = 0.35). While the downregulation of *GRIA1* is only marginally significant (*p* = 0.071), this is likely due to the small cohort size (8 schizophrenia samples and 8 controls). This explanation is further supported by the positive correlation observed with *GRIA3* expression (Fig. 4).

### 3.3 Coordinated Expression of GRIA1-3 Across Brain Tissue and Schizophrenia-Derived Organoids

To examine the consistency of transcriptional relationships among the *GRIA* genes, we computed Pearson correlation coefficients for all pairwise gene combinations within each dataset, restricted to schizophrenia samples.

In postmortem brain datasets, all *GRIA* gene pairs demonstrated strong positive correlation (**Supplementary Fig. 1**). For example, the correlation between *GRIA1* and *GRIA2* expression = 0.74, *p*-value = 3.7 × 10^-25^ (Fig. 4). A similar analysis was performed on the organoids dataset GSE133534 [65] and revealed coordinated expression patterns among *GRIA1-3*, whereas *GRIA4* did not exhibit comparable correlation with the other members of the gene family (**Supplementary Fig. 2**). Because genes involved in the same molecular pathway, such as the AMPA receptor subunits, are typically co-regulated to maintain functional integrity [83], the observation of reproducible positive correlations for *GRIA1-3* across both adult brain and early neurodevelopmental models reinforces the robustness of our findings and suggests that the observed patterns are unlikely to reflect technical artifacts or random variation.

### 3.4 Expression of GRIA Genes Shows a Significant Positive Correlation With NMDA Receptor–Encoding Genes in Postmortem Schizophrenia Brain Samples

**Fig. 5.**
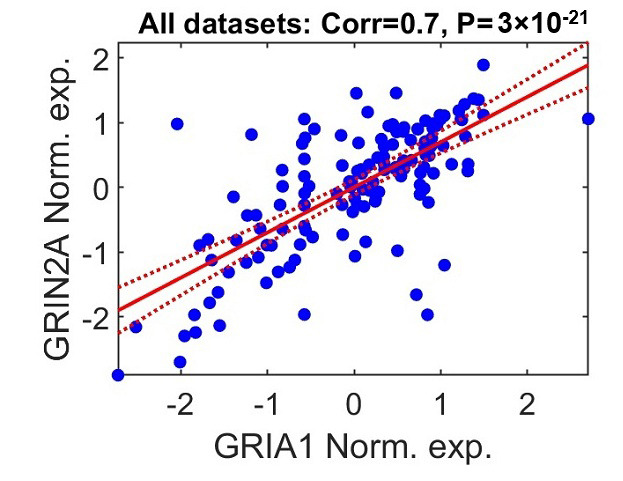
***GRIA1* and *GRIN2A* expression patterns are positively correlated**. The scatter plot illustrates the relationship between log_2_-normalized expression values of *GRIN2A* and *GRIA1* in postmortem brain samples from individuals with schizophrenia. Each point represents a single participant aggregated across the seven datasets included in the meta-analysis. Gene expression values were independently standardized within each dataset (z-scored: mean = 0, standard deviation = 1). The solid red line indicates the fitted linear regression model, and the dashed lines depict the 95% confidence interval for the regression estimate.

We further examined whether *GRIA1-4* have positively correlated expression with NMDA receptor genes. As can be seen in **Supplementary Fig. 1**, several NMDA receptor genes are positively correlated with *GRIA1-GRIA4*. For example, Glutamate Ionotropic Receptor NMDA Type Subunit 2A (*GRIN2A*), encoding NMDA receptor subunit 2A, is positively correlated with *GRIA1* (Fig. 5; combined data corr. = 0.7, *p*-value = 3 × 10^-21^).

### 3.5 Evaluation of Confounding Variables

To assess whether clinical or technical variables might influence the expression results, we constructed a linear regression model that included age, sex, pH, PMI, and antipsychotic exposure as covariates for each of the seven datasets (see Methods). Summary outcomes of this analysis are reported in **Supplementary Table 1**. However, data on these factors were not available for all datasets, and information on lifetime antipsychotic treatment was specifically available only for the parietal cortex dataset [70] (GSE35978). To summarize the linear regression analysis, the mean t-statistic values were calculated for each of the four *GRIA* genes. A consistent downward trend in expression remained even after adjusting for these confounding variables (**Supplementary Table 1**; mean *t*-statistic = –1.4, –0.71, –0.35, –1.04, respectively).

### 3.6 Per-Sample Fold Change Analysis

**Fig. 6.**
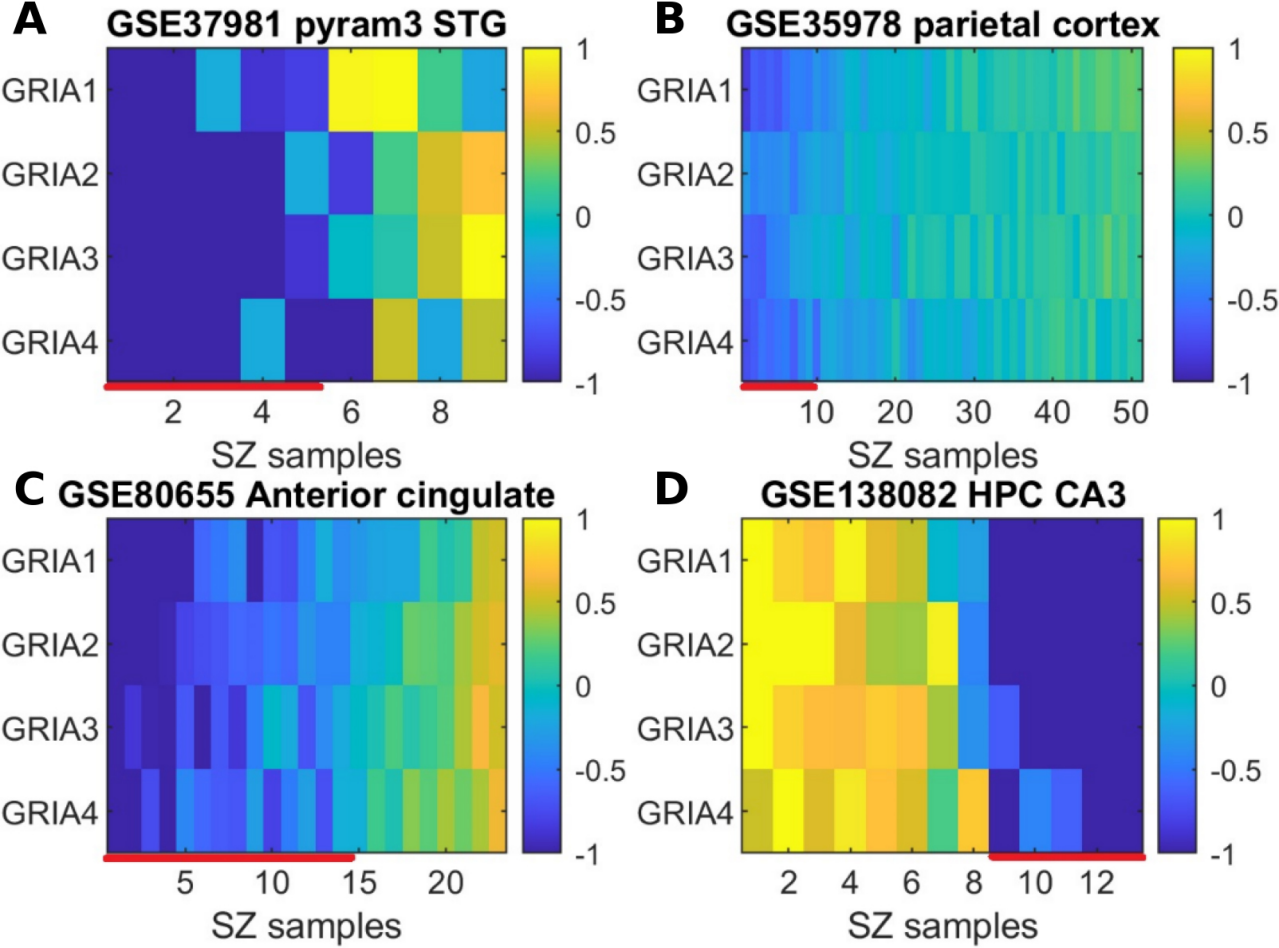
**Log_2_ fold change per-sample analysis: subgroup-specific reduction in *GRIA1-4* expression**. (A–D) display per-sample expression patterns for each dataset, with the corresponding brain region and dataset identifier indicated in the title. Within each heatmap, rows represent individual *GRIA* genes and columns correspond to schizophrenia samples. The color intensity at position (i, j) reflects the log_2_ fold change of gene *i* in patient *j*, calculated relative to the average expression in the control group. Samples exhibiting downregulation across *GRIA* genes, with mean log_2_ fold change values <–0.25, are highlighted by red bars along the x-axis to denote this subgroup.

To determine whether the observed reduction in expression is consistent across individuals or confined to a subset of individuals, we conducted a per-sample log_2_ fold-change analysis. As illustrated in Fig. 6, across four of the seven datasets, the decrease in expression of *GRIA1-4* appears to be restricted to a distinct subgroup of patients. Individuals exhibiting coordinated reduction (represented by bluish values) with mean log_2_ fold change values <–0.25 are highlighted with a red bar along the x-axis. In contrast, the remaining patients show no uniform pattern of reduction, and some display evidence of increased expression (yellow shades). This subgroup-specific pattern was replicated in the majority of datasets examined (**Supplementary Fig. 3**), supporting the notion that the downregulation is not uniformly present across the schizophrenia cohort.

To examine whether the down-regulation signal was linked to specific clinical characteristics of the patients, we calculated Pearson correlations between *GRIA* genes’ expression and the available patient variables: age, sex, and antipsychotic treatment and suicide status (treatment and suicide status were available only in one dataset). No significant associations were observed (*GRIA1* combined *p*-values = 0.3, 0.27, 0.71, 0.66, respectively; **Supplementary Figs. 4–7**; similar results were observed for *GRIA2-4*).

## 4. Discussion

In this study, we conducted a participant- level meta-analysis of the expression of *GRIA1-4* genes in brain samples from individuals with schizophrenia compared to healthy controls, by integrating seven independent gene expression datasets (a total of 295 samples, 151 with schizophrenia and 144 controls). Our analysis detects significant downregulation of the four genes encoding AMPA receptors, *GRIA1-4*, in schizophrenia. Additionally, our reanalysis of the organoid dataset from [65] confirmed downregulation of *GRIA1-3* in schizophrenia-derived organoids (*p* = 0.071, 0.005, 0.023, respectively), while *GRIA4* showed no significant differential expression (*p* = 0.35) (Fig. 3). The positive correlation between their expression patterns in both brain and organoids (Fig. 4) further supports the validity of these results and reduces the likelihood that this signal results from technical or arbitrary noise.

Genes of the *GRIA* family, which encode subunits of the AMPA glutamate receptor, play essential roles in synaptic plasticity, learning, and memory, functions strongly implicated in the cognitive and negative symptoms of schizophrenia [27, 28]. Disruption of these systems may aggravate cognitive symptoms in schizophrenia. Downregulation of these genes may impair glutamate transmission, potentially affecting a wide range of cognitive and neurological functions.

Furthermore, we found that *GRIA* genes have a positive correlation in expression with several NMDA receptor genes, including *GRIN2A*. *GRIN2A*, along with *GRIA3*, was included in the list of ten genes with ultra-rare variants associated with schizophrenia in the SCHEMA consortium study [36]. NMDA receptors are glutamate receptors involved in the development and course of both psychotic and negative symptoms in schizophrenia [14, 15, 16]. A linked regulation of genes encoding glutamate receptors, such as *GRIA* and *GRIN*, may suggest a higher-level dysregulation of glutamatergic signaling in schizophrenia. This mutual regulation could be important in understanding the complex interactions of molecular components that lead to the disease’s pathophysiology.

We note that our findings for *GRIA1-4* do not align with several previous studies that reported up-regulation in brain samples from individuals with schizophrenia. For example, Kimoto *et al*. [84] found *GRIA1* and *GRIA4* to be up-regulated in samples from 36 individuals with schizophrenia compared to 26 controls. In contrast, Beneyto and Meador-Woodruff [44] observed downregulation of *GRIA4*, consistent with our findings, but did not detect significant differential expression of *GRIA1* between 15 individuals with schizophrenia and 15 controls. One possible explanation for these inconsistencies is that *GRIA1-4* is downregulated in a subgroup of individuals with schizophrenia, and the results depend on the proportion of this subgroup within each study. Supporting this hypothesis, our per-sample fold-change analysis showed that downregulation is concentrated in a specific subgroup of patients across most datasets in our meta-analysis (Fig. 6; **Supplementary Fig. 2**). Identifying heterogeneity in gene expression patterns is essential for advancing precision medicine in schizophrenia. Stratifying patients based on molecular signatures may enable more targeted therapeutic approaches and ultimately improve treatment response and clinical outcomes. Our results indicate no substantial heterogeneity attributable to differences in brain regions or measurement platforms (Table 2; I^2^ = 0%).

### Limitations

We recognize that our study, like other postmortem studies, is limited by several factors. This postmortem examination provides a snapshot of neurobiology at the end of life and cannot detect abnormalities that may have arisen when the disease first manifested itself. This is especially important in the case of schizophrenia, as research suggests that its pathophysiology develops in its early stages [85]. Additionally, the observed differential expression may be influenced by confounding factors, such as medication and illness duration. While our linear regression analyses suggest that the observed downregulation cannot be explained solely by age or sex, several potential confounders were not fully assessed. Information on PMI and pH was incomplete across studies, antipsychotic medication data were available for only one dataset, and duration of illness was not reported. Therefore, we cannot exclude the possibility that these or other confounding factors contribute to the observed downregulation.

However, the study of schizophrenia iPSCs-derived cerebral organoids, which represent early human brain development [65] and our re-analysis (Figs. 3,4), suggests that the downregulation of *GRIA1-3* is already present in the early stages of the disease. Furthermore, the significant correlation we observe between *GRIA* genes’ expression, in both brain and organoid samples (**Supplementary Fig. 1,2**), provides additional validation for its downregulation in schizophrenia. Although *GRIA1-3* showed consistent downregulation across both postmortem brain tissue and organoids, *GRIA4* did not demonstrate differential expression in the developmental model. This discrepancy may indicate temporal heterogeneity in AMPA receptor subunit vulnerability, where *GRIA4* changes either emerge later in neurodevelopment or are restricted to a more specific clinical subgroup. Alternatively, *GRIA4* dysregulation may require environmental or pharmacological exposure not captured in early organoid models. Together, these patterns support the interpretation that *GRIA1-3* represent a core early neurodevelopmental AMPAR signature, while *GRIA4* may participate in a downstream or secondary phase of disease-related synaptic remodeling.

Another limitation is that transcript levels do not necessarily reflect protein abundance or receptor functionality, and caution is required in interpreting downstream physiological implications. Future studies integrating proteomic, electrophysiological, and functional readouts are required for clarifying the biological consequences of altered *GRIA* expression in schizophrenia.

In summary, our analysis identifies a subgroup of individuals with schizophrenia showing coordinated downregulation of *GRIA1-4*, which encodes AMPA receptor subunits. The identification of distinct molecular subgroups is an essential step toward the development of personalized medicine in schizophrenia. The downregulation of *GRIA1-3* in organoid models suggests that these changes arise before disease onset. Combined with the established genetic link between *GRIA* genes and schizophrenia, as well as the critical role of glutamate signaling in its pathophysiology, our findings indicate that this differential expression may contribute to disease development.

This research underscores the need for further investigation into AMPA receptor modulation as a potential biomarker and therapeutic target for a distinct subgroup of patients with schizophrenia. Such insights could pave the way for the development of personalized treatment approaches.

## 5. Conclusions

*GRIA1-4* are significantly downregulated in a subgroup of individuals with schizophrenia, with changes in *GRIA1-3* already evident in cerebral organoids, suggesting an early neurodevelopmental origin. While no clear clinical characteristics were found to distinguish this subgroup, these findings indicate a distinct molecular subgroup of patients and highlight *GRIA* expression as a potential biomarker and therapeutic target for advancing personalized treatment in schizophrenia.

## Data Availability

All datasets analyzed in this study were previously published and are publicly accessible through the Gene Expression Omnibus (GEO) database (https://www.ncbi.nlm.nih.gov/geo/).

## Abbreviations

ACC, anterior cingulate cortex; AMPA, α-amino-3-hydroxy-5-methyl-4-isoxazole propionic acid; AMPARs, AMPA receptors; BA, brodmann area; CA3, Cornu Ammonis 3; CC, cingulate cortex; CCHPC, Charing Cross Hospital Prospective Collection; CNT, controls; CRBLM, cerebellum; GEO, Gene Expression Omnibus; GRIA, Glutamate Ionotropic Receptor AMPA Type Subunit; HBTRC, The Harvard Brain Tissue Resource Center; HPC, hippocampus; iPSCs, Induced Pluripotent Stem Cells; NAcc, Nucleus Accumbens; NMDA, N-methyl-D-aspartate; NMDARs, NMDA receptors; PMI, postmortem interval; PRISMA, Preferred Reporting Items for Systematic Reviews and Meta-Analyses; SMRI, Stanley Medical Research Institute; STG, Superior Temporal Gyrus; SZ, schizophrenia.

## Supplementary Material

Supplementary material associated with this article can be found, in the online version, at https://doi.org/10.31083/AP46200.
