# Supplementary material for: GRIA Gene Expression in Schizophrenia: A Participant-Level Meta-Analysis
Source: Alpha Psychiatry. 2026 May 21;27(3):46200. doi: 10.31083/AP46200 (PMC13339803; doi:10.31083/AP46200)
Supplement: Supplementary file 1 [file 2757-8038-27-3-46200-s1.zip › Supplementary Material.docx]

GRIA genes encoding the glutamate AMPA receptor are down-regulated in a subgroup of individuals with schizophrenia

SUPPLEMENTARY INFORMATION

CONTENTS

[LIST OF FIGURES 2](#_Toc225756911)

[LIST OF TABLES 3](#_Toc225756912)

[BRAIN SAMPLES DATASETS CHARACTERISTICS 4](#_Toc225756913)

[**Maycox 2009 BA10 dataset** 4](#_Toc225756914)

[**Pietersen 2014 Pyramidal STG dataset** 4](#_Toc225756915)

[**Paz 2006 cerebellum dataset** 5](#_Toc225756916)

[**Chen 2013 parietal cortex dataset** 5](#_Toc225756917)

[**Ramaker 2017 anterior cingulate gyrus dataset** 6](#_Toc225756918)

[**Lanz 2019 hippocampus dataset** 6](#_Toc225756919)

[**Perez 2021 hippocampal subfield CA3 dataset** 7](#_Toc225756920)

[INDUCED PLURIPOTENT CELLS (iPSCs)-DERIVED DATASETS CHARACTERISTICS 8](#_Toc225756921)

[**Kathuria 2020 iPSC-derived cerebral organoid dataset** 8](#_Toc225756922)

[SUPPLEMENTARY FIGURES AND TABLES 9](#_Toc225756923)

[BIBLIOGRAPHY 15](#_Toc225756924)

# LIST OF FIGURES

[Supplementary Fig. 1. -Log2 of p-values of Pairwise Pearson correlation of GRIA1-4 and GRIN2A-D genes along schizophrenia brain samples in the datasets included in the meta-analysis. The color in each entry (x,y) represents the –Log2 of the p-value associated with the Pearson correlation coefficient between the expression of the gene in row x and the gene in column y, measured along the schizophrenia samples. Gene symbols are ordered in the same order in both the x- and the y-axes. 11](#_Toc227408778)

[Supplementary Fig. 2. -Log2 of p-values of Pairwise Pearson correlation of GRIA1-4 expression along schizophrenia samples organoids. The color in each entry (x,y) represents the –Log2 of the p-value associated with the Pearson correlation coefficient between the expression of the gene in row x and the gene in column y, measured along the schizophrenia samples. Gene symbols are ordered in the same order in both the x- and the y-axes. 12](#_Toc227408779)

[Supplementary Fig. 3. Brain samples per-sample Log2 fold change analysis. Each plot represents a gene expression dataset, where the brain region and dataset ID are specified in the title. In each plot, each row represents a gene and each column represents a patient with schizophrenia. The color in entry (i,j) represents the Log2 fold change (expression of gene i in patient j, divided by its mean expression among the controls). Subgroups of patients with a down-regulation pattern of these genes (bluish fold change values colors) are marked with a red line along the x-axes. 12](#_Toc227408780)

[Supplementary Fig. 4. Correlation between GRIA1 Log2 expression and the age of the patients. For each of the datasets, a scatter plot of GRIA1 Log2 expression (x-axis) and the age of the patients with schizophrenia (y-axis) is presented. Each point represents the expression in one individual with schizophrenia. The red line represents the linear regression line. The dashed-red lines represent 95% confidence bands. Pearson correlation values and the associated p-values are written in the title of each subplot. Pearson correlation is plotted also for a union of the datasets, where the genes’ expression levels were normalized in each of the datasets separately (mean: 0; standard deviation: 1) 13](#_Toc227408781)

[Supplementary Fig. 5. Correlation between GRIA1 Log2 expression and the gender of the patients. For each of the datasets, a scatter plot of GRIA1 Log2 expression (x-axis) and the gender of the patients with schizophrenia (y-axis) is presented. Each point represents the expression in one individual with schizophrenia. The red line represents the linear regression line. The dashed-red lines represent 95% confidence bands. Pearson correlation values and the associated p-values are written in the title of each subplot. Pearson correlation is plotted also for a union of the datasets, where the genes’ expression levels were normalized in each of the datasets separately (mean: 0; standard deviation: 1) 14](#_Toc227408782)

[Supplementary Fig. 6. Correlation between GRIA1 Log2 expression and the suicide of the patients. For the single dataset for which this information was available, a scatter plot of GRIA1 Log2 expression (x-axis) and the suicide status of the patients with schizophrenia (y-axis) is presented. Each point represents the expression in one individual with schizophrenia. The red line represents the linear regression line. The dashed-red lines represent 95% confidence bands. Pearson correlation values and the associated p-values are written in the title of each subplot. 15](#_Toc227408783)

[Supplementary Fig. 7. Correlation between GRIA1 Log2 expression and the Lifetime antipsychotic treatment of the patients. For the single dataset for which this information was available, a scatter plot of GRIA1 Log2 expression (x-axis) and the lifetime antipsychotic treatment (measured as quantity of Fluphenazine or equivalent anti-psychotic given over a lifetime, in mg) of the patients with schizophrenia (y-axis) is presented. Each point represents the expression in one individual with schizophrenia. The red line represents the linear regression line. The dashed-red lines represent 95% confidence bands. Pearson correlation values and the associated p-values are written in the title of each subplot. 15](#_Toc227408784)

# LIST OF TABLES

[Table 1S. Linear model t-statistic values and associated p-values for each of the seven datasets, for GRIA1-4. T-statistic values higher than zero are marked red and t-statistic values lower than zero are marked blue. The color intensity is proportional to the t-statistic value. The p-value associated with each t-statistic value appears in the row below the t-statistic value. 14](#_Toc176169197)

# BRAIN SAMPLES DATASETS CHARACTERISTICS

## **Maycox 2009 BA10 dataset**

The dataset GDS4523 (Maycox et al., 2009) was downloaded from the GEO database (<https://www.ncbi.nlm.nih.gov/sites/GDSbrowser?acc=GDS4523>). The dataset consists of 51 BA10 brain samples from subjects with schizophrenia (n=28) and healthy controls (n=23). See Figure 2 for samples’ characteristics. Samples were run on Affymetrix Human Genome U133 Plus 2.0 Arrays. **Normalization method:** Arrays were scanned on a GeneChip Scanner 3000 and fluorescence intensity for each feature of the array was obtained by using GeneChip Operating Software (Affymetrix) (Maycox et al., 2009). As described in GSE17612_series_matrix.txt (available at <https://www.ncbi.nlm.nih.gov/geo/query/acc.cgi?acc=GSE17612>), the data were analyzed with Microarray Suite version 5.0 (MAS 5.0) using Affymetrix default analysis settings and global scaling as normalization method. The trimmed mean target intensity of each array was set to 150. We then applied threshold and log2. The threshold value was determined using scatter plots of healthy control samples in order to estimate the noise level (the threshold after log2 that was used is 4). **Outlier removal:** Outlier samples removal was applied similarly to (Oldham et al., 2008). Shortly, the following steps were applied: 1) Pearson correlation coefficient values between each pair of samples were calculated. 2) For each sample, the mean correlation with all other sample was calculated. 3) The mean, M, and standard deviation, S, of all samples’ mean values (calculated in 2.) were calculated. 4) Samples whose mean correlation (found in 2.) were smaller than M-3*S were considered as outliers and were removed from the rest of the analysis. One schizophrenia sample was removed. After outlier removal, 50 samples were left (27 schizophrenia and 23 controls). **Filtering**: The initial number of probe-sets was 54,613. 1) In case of a gene symbol with multiple probe-sets, the probe-set with the highest mean expression over the samples was taken into account and the other probe-sets were discarded. Number of genes after this step: 30,805. 2) Genes that were absent (values equal or lower than the threshold) in more than 70% of both the schizophrenia and the control samples, were filtered out. Number of genes after filtering: 27,261.

##

## **Pietersen 2014 Pyramidal STG dataset**

The dataset GSE37981 (Pietersen et al., 2014) was downloaded from the GEO database (<https://www.ncbi.nlm.nih.gov/geo/query/acc.cgi?acc=GSE37981>). The initial dataset consists of 18 brain samples isolated from pyramidal cells in layer III of the superior temporal gyrus (STG) with schizophrenia (n=9) and healthy controls (n=9). The samples were run on Affymetrix Human X3P Array. **Normalization method:** “Each array was scanned twice and the Affymetrix Microarray Suite 5.1 software averaged the two images to compute an intensity value for each probe cell within each probe set” (Pietersen et al., 2014).

We then applied threshold and log2. The threshold value was determined using scatter plots of healthy control samples in order to estimate the noise level (the threshold after log2 that was used is 3).  **Outlier removal:** Outlier samples removal was applied similarly to (Oldham et al., 2008) and as described above. After outlier removal, 1 normal sample was removed. After the removal, the dataset consists of 17 samples (9 schizophrenia and 8 controls). **Filtering**: Initial number of probe-sets: 61,359 (48,860 with gene symbols). 1) In case of a gene symbol with multiple probe-sets, the probe-set with the highest mean expression over the samples was taken into account and the other probe-sets were discarded. Number of genes after this step: 21,606. 2) Genes that were absent (values equal or lower than the threshold) in more than 70% of both the schizophrenia and the control samples, were filtered out. Number of genes after filtering: 17,886.

## **Paz 2006 cerebellum dataset**

The dataset GDS1917 (Paz et al., 2006) was downloaded from the GEO database (<https://www.ncbi.nlm.nih.gov/sites/GDSbrowser?acc=GDS1917>). The initial dataset consists of 28 brain samples corresponding to the crus I/VIIa area of the cerebellum from subjects with schizophrenia (n=14) and healthy controls (n=14) (The samples were obtained from the Maryland Brain Collection). The samples were run on Affymetrix Human Genome U133 Plus 2.0 Array. **Normalization method:** We applied threshold and Log2 transformation. The threshold value was determined using scatter plots of healthy control samples in order to estimate the noise level (the threshold after Log2 that was used is 4). **Outlier removal:** Outlier samples removal was applied similarly to (Oldham et al., 2008). Shortly, the following steps were applied: 1) Pearson correlation coefficient values between each pair of samples were calculated. 2) For each sample, the mean correlation with all other sample was calculated. 3) The mean, M, and standard deviation, S, of all samples’ mean values (calculated in 2.) were calculated. 4) Samples whose mean correlation (found in 2.) were smaller than M-3*S were considered as outliers and were removed from the rest of the analysis. One schizophrenia sample was removed. After the removal, 27 samples were left (13 schizophrenia and 14 controls). **Filtering**: The initial number of probe-sets was 54,613. 1) In case of a gene symbol with multiple probe-sets, the probe-set with the highest mean expression over the samples was taken into account and the other probe-sets were discarded. Number of genes after this step: 30,803. 2) Genes that were absent (values equal or lower than the threshold) in more than 70% of both the schizophrenia and the control samples, were filtered out. Number of genes after filtering: 27,329.

##

## **Chen 2013 parietal cortex dataset**

The dataset GSE35978 (Chen et al., 2013) was downloaded from the GEO database (<https://www.ncbi.nlm.nih.gov/geo/query/acc.cgi?acc=GSE35978>). The initial dataset consisted of 312 brain samples, including subjects with schizophrenia (n=51) and healthy controls (n=50) from the parietal cortex. Samples were run on Affymetrix Human Gene 1.0 ST Array [transcript (gene) version]. **Normalization method:** “The data were analyzed by RMA using Affymetrix Expression Console with default analysis settings” (described in GSE35978_series_matrix.txt, available at the aforementioned link). After applying RMA, no threshold or log2 are needed. That is because the RMA normalization method includes the following steps: 1. Background correction 2. Quantile normalization 3. Log2 transformation.

**Outlier removal:** Outlier samples removal was applied similarly to (Oldham et al., 2008) and as described above. (**Note**: this procedure was performed on the initial dataset of 312 samples). 5 control samples were removed. After the removal, the dataset consists of 51 schizophrenia samples and 45 healthy control samples. **Filtering**: The initial number of probe-sets was 33,297 (25,293 with gene symbols). In case of a gene symbol with multiple probe-sets, the probe-set with the highest mean expression over the samples (of both brain regions) was taken into account and the other probe-sets were discarded. Number of genes after this step: 23,307.

##

## **Ramaker 2017 anterior cingulate gyrus dataset**

The dataset GSE80655 (Ramaker et al., 2017) was downloaded from the GEO database (<https://www.ncbi.nlm.nih.gov/geo/query/acc.cgi?acc=GSE80655>). The initial dataset consisted of 281 brain samples, including subjects with schizophrenia (n=24) and healthy controls (n=24) from the anterior cingulate gyrus (AnCg). Samples were run on Illumina HiSeq 2000 (Homo sapiens). **Normalization method:** “RNA-seq paired-end FASTQ files were aligned to hg19 using Tophat and Cufflinks. Read counts for each transcript were compiled across all post-mortem tissues samples” (described in GSE80655_series_matrix.txt, available at the aforementioned link). We then applied threshold and log2. The threshold value was determined using scatter plots of healthy control samples in order to estimate the noise level (the threshold after log2 that was used is 3). **Outlier removal:** Outlier samples removal was applied similarly to (Oldham et al. 2008) and as described above. (**Note:** this procedure was performed on the initial dataset of 281 samples). 1 schizophrenia sample was removed. After the removal, the dataset consists of 23 schizophrenia samples and 24 healthy control samples. **Filtering**: Initial number of probe-sets: 57,905 (52,442 with gene symbols). 1) In case of a gene symbol with multiple probe-sets, the probe-set with the highest mean expression over the samples (of both brain regions) was taken into account and the other probe-sets were discarded. Number of genes after this step: 51,310. 2) Genes that were absent (values equal or lower than the threshold) in more than 70% of both the schizophrenia and the control samples, were filtered out. Number of genes after filtering: 23,175.

##

## **Lanz 2019 hippocampus dataset**

The dataset GSE53987 (Lanz et al. 2019) was downloaded from the GEO database (<https://www.ncbi.nlm.nih.gov/geo/query/acc.cgi?acc=GSE53987>). The initial dataset consisted of 205 brain samples, including subjects with schizophrenia (n=15) and healthy controls (n=18) from the hippocampus. Samples were run on Affymetrix Human Genome U133 Plus 2.0 Arrays. **Normalization method:** “R/Bioconductor was used to apply the Robust Multi-Array Average (RMA) methodology to generate expression values. Brain regions were normalized separately” (described in GSE53987_series_matrix.txt, available at the aforementioned link). We then applied log2. **Outlier removal:** Outlier samples removal was applied similarly to (Oldham et al., 2008) and as described above. (**Note**: this procedure was performed on the initial dataset of 205 samples). No sample was removed. **Filtering**: The initial number of probe-sets was 54,613 (45,772 with gene symbols). In case of a gene symbol with multiple probe-sets, the probe-set with the highest mean expression over the samples (of both brain regions) was taken into account and the other probe-sets were discarded. Number of genes after this step: 22,880.

## **Perez 2021 hippocampal subfield CA3 dataset**

The dataset GSE138082 (Perez et al. 2021) was downloaded from the GEO database (<https://www.ncbi.nlm.nih.gov/geo/query/acc.cgi?acc=GSE138082>). The initial dataset consisted of 78 brain samples, including subjects with schizophrenia (n=39 (13 samples from CA3)) and healthy controls (n=39 (13 from CA3)). Samples were run on Illumina HiSeq 2500 Arrays. **Normalization method:** Single-end/Paired-end reads were aligned with STAR 2.5.2b to the reference sequence (hg19 from UCSC). Human reference was used for gene annotation and counting (GRCh37.87). Uniquely mapped reads were quantified using HTSeq python suite using protein coding genes by gene (described in GSE138082_series_matrix.txt, available at the aforementioned link). We then applied threshold and log2. The threshold value was determined using scatter plots of healthy control samples in order to estimate the noise level (the threshold after log2 that was used is 5). **Outlier removal:** Outlier samples removal was applied similarly to (Oldham et al. 2008) and as described above. (**Note**: this procedure was performed on the initial dataset of 78 samples). One control sample (CA3) was removed. After the removal, the dataset consists of 13 schizophrenia samples and 12 healthy control samples from CA3. **Filtering:** The initial number of gene symbols was 20,268. Genes that were absent (values equal or lower than the threshold) in more than 70% of both the schizophrenia and the control samples in CA3, were filtered out. Number of genes after filtering: 14,571.

#

# INDUCED PLURIPOTENT CELLS (iPSCs)-DERIVED DATASETS CHARACTERISTICS

## **Kathuria 2020 iPSC-derived cerebral organoid dataset**

The dataset GSE133534 (Kathuria et al., 2020) was downloaded from the GEO database (<https://www.ncbi.nlm.nih.gov/geo/query/acc.cgi?acc=GSE133534>). The initial dataset consists of 21 induced Pluripotent Stem Cell (iPSC) derived cerebral organoids samples with schizophrenia (n=10) and healthy controls (n=11). Samples were run on Illumina NovaSeq 6000 (Homo sapiens). **Normalization method:** Sequence quality was assessed using FASTQC. Sequences were trimmed using cutadapt v 1.18. Trimmed reads were aligned to the reference genome using TopHat v2.1.1. Bam files were merged using samptools v 1.9 for samples with techincal replicates. FPKM calculation done using cufflinks v2.2.1. (described in GSE133534_series_matrix.txt, available at the aforementioned link). After merging the replicates, the dataset consists of 16 samples (8 schizophrenia and 8 controls). We then applied threshold and log2. The threshold value was determined using scatter plots of healthy control samples in order to estimate the noise level (the threshold after log2 that was used is log2(0.008)). **Outlier removal:** Outlier samples removal was applied similarly to (Oldham et al., 2008) and as described above. No sample was removed. **Filtering**: The initial number of gene symbols was 49,554. 1) In case of identical symbols, the symbol with the highest mean expression over the samples was taken into account and the other identical symbols were discarded. Number of gene symbols after this step: 45,331. 2) Genes that were absent (values equal or lower than the threshold) in more than 70% of both the schizophrenia and the control samples, were filtered out. Number of gene symbols after filtering: 34,302.

# SUPPLEMENTARY FIGURES AND TABLES


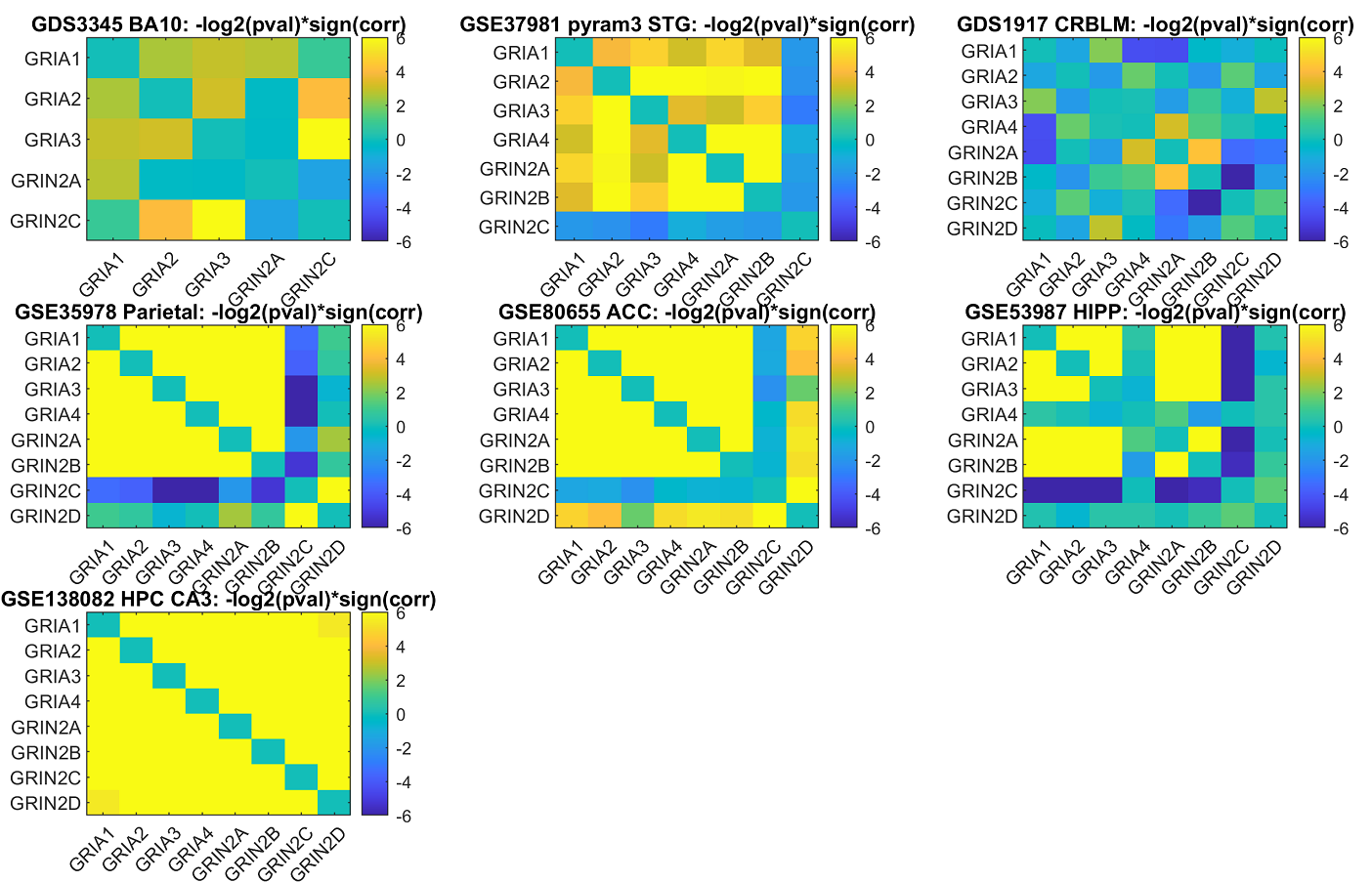
**Supplementary Fig. 1. -Log2 of p-values of Pairwise Pearson correlation of GRIA1-4 and GRIN2A-D genes along schizophrenia brain samples in the datasets included in the meta-analysis.** The color in each entry (x,y) represents the –Log2 of the p-value associated with the Pearson correlation coefficient between the expression of the gene in row x and the gene in column y, measured along the schizophrenia samples. Gene symbols are ordered in the same order in both the x- and the y-axes.

**Supplementary Fig. 2. -Log2 of p-values of Pairwise Pearson correlation of GRIA1-4 expression along schizophrenia samples organoids.** The color in each entry (x,y) represents the –Log2 of the p-value associated with the Pearson correlation coefficient between the expression of the gene in row x and the gene in column y, measured along the schizophrenia samples. Gene symbols are ordered in the same order in both the x- and the y-axes.


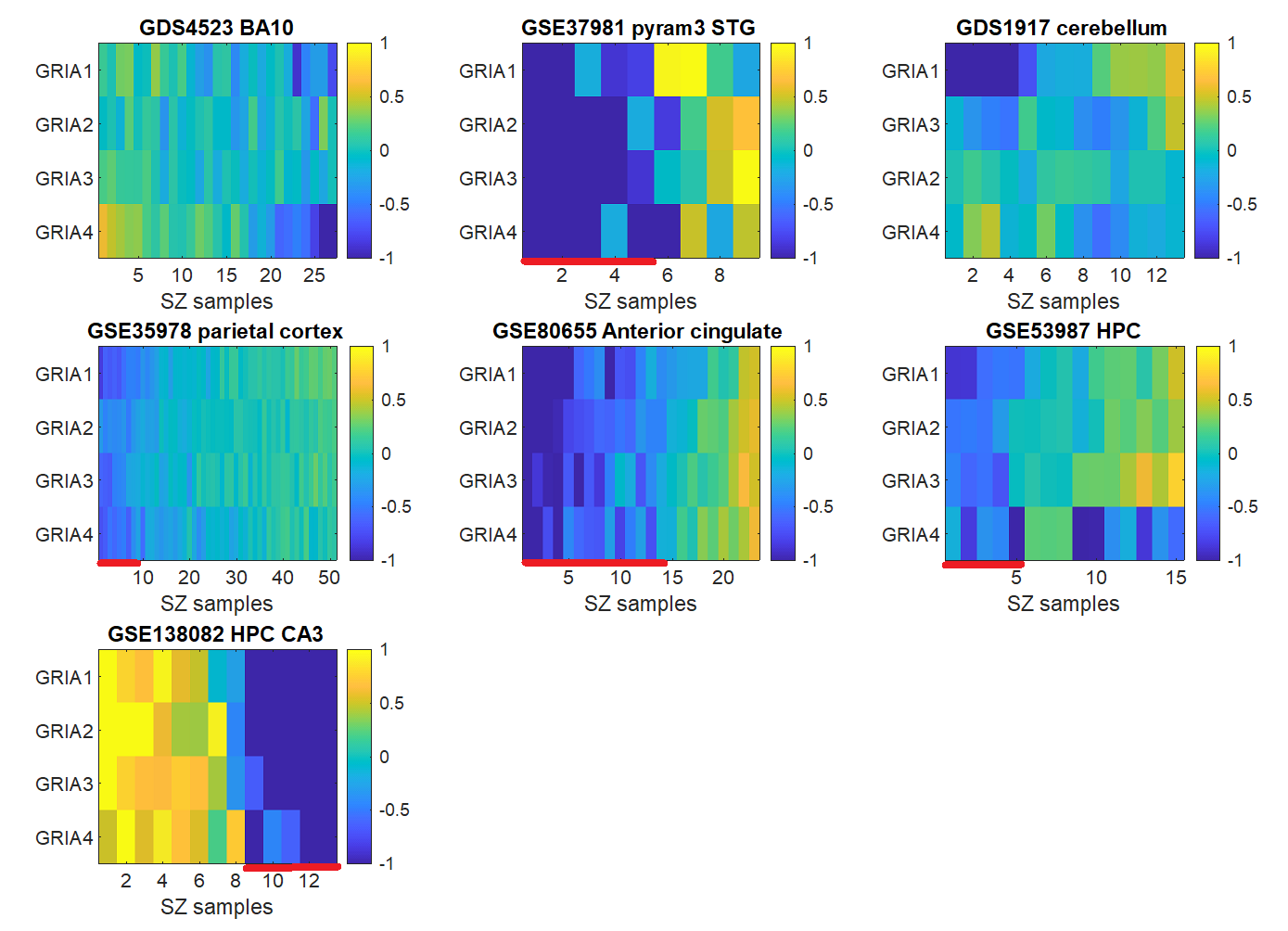


Supplementary Fig. 3. Brain samples per-sample Log2 fold change analysis. Each plot represents a gene expression dataset, where the brain region and dataset ID are specified in the title. In each plot, each row represents a gene and each column represents a patient with schizophrenia. The color in entry (i,j) represents the Log2 fold change (expression of gene i in patient j, divided by its mean expression among the controls). Subgroups of patients with a down-regulation pattern of these genes (bluish fold change values colors) are marked with a red line along the x-axes.


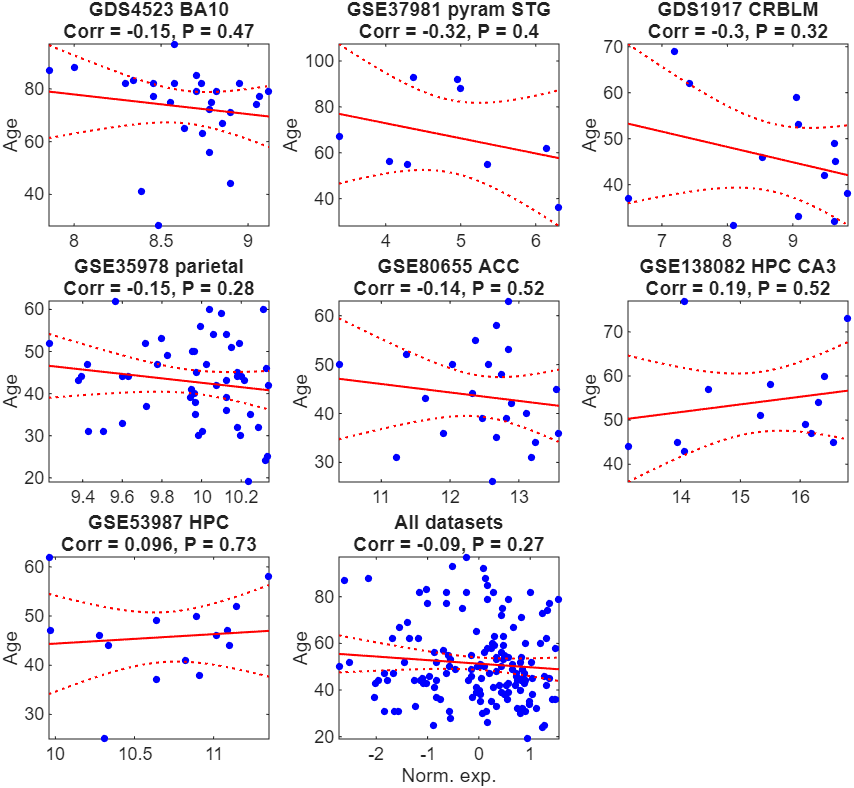


Supplementary Fig. 4. Correlation between GRIA1 Log2 expression and the age of the patients. For each of the datasets, a scatter plot of GRIA1 Log2 expression (x-axis) and the age of the patients with schizophrenia (y-axis) is presented. Each point represents the expression in one individual with schizophrenia. The red line represents the linear regression line. The dashed-red lines represent 95% confidence bands. Pearson correlation values and the associated p-values are written in the title of each subplot. Pearson correlation is plotted also for a union of the datasets, where the genes’ expression levels were normalized in each of the datasets separately (mean: 0; standard deviation: 1)


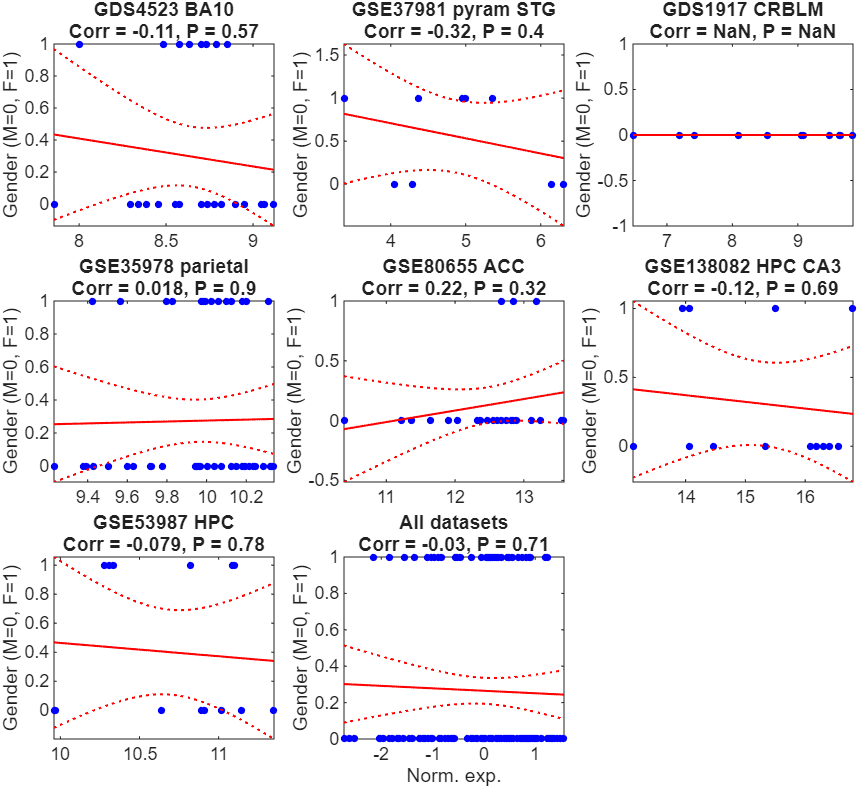
 Supplementary Fig. 5. Correlation between GRIA1 Log2 expression and the gender of the patients. For each of the datasets, a scatter plot of GRIA1 Log2 expression (x-axis) and the gender of the patients with schizophrenia (y-axis) is presented. Each point represents the expression in one individual with schizophrenia. The red line represents the linear regression line. The dashed-red lines represent 95% confidence bands. Pearson correlation values and the associated p-values are written in the title of each subplot. Pearson correlation is plotted also for a union of the datasets, where the genes’ expression levels were normalized in each of the datasets separately (mean: 0; standard deviation: 1)


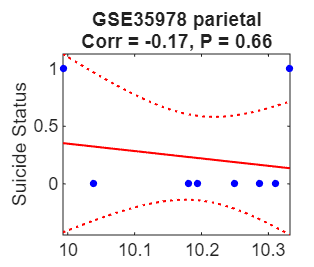


Supplementary Fig. 6. Correlation between GRIA1 Log2 expression and the suicide of the patients. For the single dataset for which this information was available, a scatter plot of GRIA1 Log2 expression (x-axis) and the suicide status of the patients with schizophrenia (y-axis) is presented. Each point represents the expression in one individual with schizophrenia. The red line represents the linear regression line. The dashed-red lines represent 95% confidence bands. Pearson correlation values and the associated p-values are written in the title of each subplot.


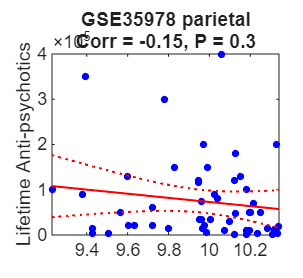


Supplementary Fig. 7. Correlation between GRIA1 Log2 expression and the Lifetime antipsychotic treatment of the patients. For the single dataset for which this information was available, a scatter plot of GRIA1 Log2 expression (x-axis) and the lifetime antipsychotic treatment (measured as quantity of Fluphenazine or equivalent anti-psychotic given over a lifetime, in mg) of the patients with schizophrenia (y-axis) is presented. Each point represents the expression in one individual with schizophrenia. The red line represents the linear regression line. The dashed-red lines represent 95% confidence bands. Pearson correlation values and the associated p-values are written in the title of each subplot.

Supplementary Table 1. Linear model t-statistic values and associated p-values for each of the seven datasets, for GRIA1-4. T-statistic values higher than zero are marked red and t-statistic values lower than zero are marked blue. The color intensity is proportional to the t-statistic value. The p-value associated with each t-statistic value appears in the row below the t-statistic value.

| # | Dataset | GRIA1 | GRIA2 | GRIA3 | GRIA4 | Covariates included |
| --- | --- | --- | --- | --- | --- | --- |
| 1 | GDS4523 BA10 t-stat | 0.33 | -0.38 | 1.13 | 1.30 | Age, Gender, PMI, pH |
|  | p-value | 0.74 | 0.71 | 0.27 | 0.20 |  |
| 2 | GSE37981 pyram3 STG t-stat | -0.29 | -1.10 | -0.80 | -1.43 | Age, Gender, PMI, pH |
|  | p-value | 0.78 | 0.29 | 0.44 | 0.18 |  |
| 3 | GDS1917 CRBLM t-stat | -1.81 | 0.43 | -1.13 | -1.18 | Age, Gender, PMI |
|  | p-value | 0.08 | 0.67 | 0.27 | 0.25 |  |
| 4 | GSE35978 parietal t-stat | -0.46 | -0.81 | 0.04 | -1.51 | Age, Gender, Lifetime Anti-psychotics, PMI, pH |
|  | p-value | 0.65 | 0.42 | 0.96 | 0.13 |  |
| 5 | GSE80655 ACC t-stat | -2.38 | -1.51 | -0.99 | -1.30 | Age, Gender, PMI, pH |
|  | p-value | 0.02 | 0.14 | 0.33 | 0.20 |  |
| 6 | GSE53987 HPC t-stat | -1.93 | -1.03 | -0.28 | -2.85 | Age, Gender, PMI, pH |
|  | p-value | 0.06 | 0.31 | 0.78 | 0.01 |  |
| 7 | GSE138082 HPC CA3 t-stat | -0.74 | -0.56 | -0.43 | -0.31 | Age, Gender, PMI |
|  | p-value | 0.47 | 0.58 | 0.67 | 0.76 |  |
|  | Mean t-statistic | -1.04 | -0.71 | -0.35 | -1.04 |  |

# BIBLIOGRAPHY

Chen, C., Cheng, L., Grennan, K., Pibiri, F., Zhang, C., Badner, J.A., Kelsoe, J.R., Greenwood, T.A., Nievergelt, C.M., Barrett, T.B., McKinney, R., Shilling, P.D., Schork, N.J., Smith, E.N., Bloss, C.S., Nurnberger, J., Edenberg, H.J., Foroud, T., Koller, D.L., Scheftner, W., Lawson, W.B., Nwulia, E.A., Hipolito, M., Coryell, W., Rice, J., Byerley, W., McMahon, F., Chen, D.T.W., Schulze, T.G., Berrettini, W., Potash, J.B., Zandi, P.P., Mahon, P.B., McInnis, M., Craig, D., Szelinger, S., Gershon, E.S., Liu, C., 2013. Two gene co-expression modules differentiate psychotics and controls. Mol. Psychiatry 18, 1308–1314. https://doi.org/10.1038/mp.2012.146

Kathuria, A., Lopez-Lengowski, K., Jagtap, S.S., McPhie, D., Perlis, R.H., Cohen, B.M., Karmacharya, R., 2020. Transcriptomic Landscape and Functional Characterization of Induced Pluripotent Stem Cell-Derived Cerebral Organoids in Schizophrenia. JAMA psychiatry 77, 745–754. https://doi.org/10.1001/JAMAPSYCHIATRY.2020.0196

Maycox, P.R., Kelly, F., Taylor, A., Bates, S., Reid, J., Logendra, R., Barnes, M.R., Larminie, C., Jones, N., Lennon, M., Davies, C., Hagan, J.J., Scorer, C.A., Angelinetta, C., Akbar, T., Hirsch, S., Mortimer, A.M., Barnes, T.R.E., de Belleroche, J., de Belleroche, J., 2009. Analysis of gene expression in two large schizophrenia cohorts identifies multiple changes associated with nerve terminal function. Mol. Psychiatry 14, 1083–1094. https://doi.org/10.1038/mp.2009.18

Oldham, M.C., Konopka, G., Iwamoto, K., Langfelder, P., Kato, T., Horvath, S., Geschwind, D.H., 2008. Functional organization of the transcriptome in human brain. Nat. Neurosci. 11, 1271–1282. https://doi.org/10.1038/nn.2207

Paz, R.D., Andreasen, N.C., Daoud, S.Z., Conley, R., Roberts, R., Bustillo, J., Perrone-Bizzozero, N.I., 2006. Increased Expression of Activity-Dependent Genes in Cerebellar Glutamatergic Neurons of Patients With Schizophrenia. Am. J. Psychiatry 163, 1829–1831. https://doi.org/10.1176/ajp.2006.163.10.1829

Pietersen, C.Y., Mauney, S.A., Kim, S.S., Lim, M.P., Rooney, R.J., Goldstein, J.M., Petryshen, T.L., Seidman, L.J., Shenton, M.E., McCarley, R.W., Sonntag, K.C., Woo, T.U.W., 2014. Molecular profiles of pyramidal neurons in the superior temporal cortex in schizophrenia. J. Neurogenet. 28, 53–69. https://doi.org/10.3109/01677063.2014.882918

Ramaker, R.C., Bowling, K.M., Lasseigne, B.N., Hagenauer, M.H., Hardigan, A.A., Davis, N.S., Gertz, J., Cartagena, P.M., Walsh, D.M., Vawter, M.P., Jones, E.G., Schatzberg, A.F., Barchas, J.D., Watson, S.J., Bunney, B.G., Akil, H., Bunney, W.E., Li, J.Z., Cooper, S.J., Myers, R.M., 2017. Post-mortem molecular profiling of three psychiatric disorders. Genome Med. 9, 72. https://doi.org/10.1186/s13073-017-0458-5
